# Phenotypic and molecular identification of *Sporothrix* isolates of clinical origin in Northeast China

**DOI:** 10.1007/s11046-013-9668-6

**Published:** 2013-06-16

**Authors:** Xiaohong Yu, Zhe Wan, Zhenying Zhang, Fuqiu Li, Ruoyu Li, Xiaoming Liu

**Affiliations:** 1 Department of Dermatology and Venerology, The 1st Affiliated Hospital of Dalian Medical University, No.222, Zhongshan Road, Dalian, 116011 China; 2 Department of Dermatology and Venerology, Centre for Medical Mycology and Mycoses, The First Hospital, Peking University, No.8, Xishiku Street, Xicheng District, Beijing, 100034 China; 3 Department of Dermatology and Venerology, The Second Hospital of Jilin University, No.218, Ziqiang Street, Changchun, 130041 China

**Keywords:** Sporotrichosis, *Sporothrix globosa*, Calmodulin gene, Northeast China

## Abstract

Sporotrichosis is the most common deep mycosis in Northeast China which is an area of high epidemicity due to contact with reeds or cornstalks. In this study, we have characterized a total of 74 clinical isolates from fixed cutaneous, lymphocutaneous and disseminated clinical forms and from Heilongjiang, Jilin, and Liaoning provinces, respectively. All isolates (previously as *Sporothrix schenckii*) were identified as *Sporothrix globosa* according to their phenotypic characteristics and calmodulin gene sequences analysis. They were subdivided into two sub-clades (*S. globosa* I and *S. globosa* II). Most of our isolates (71/74) presented restricted growth at 37 °C, which differed from a previous report. Up to now, *S. globosa* is the only pathogenic species in Northeast China, no matter what kind of clinical form and which region it is isolated from. Most of our clinical isolates (68/74) were clustered with three Chinese environmental isolates reported in the literature. The new findings of *S. globosa* isolates on division and thermotolerance at 37 °C described in this study will help us gain a better understanding of *S. globosa*.

## Introduction

Sporotrichosis is a common subcutaneous mycosis which is caused by the dimorphic fungus previously described as the single species *Sporothrix schenckii,* now being recognized as *Sporothrix* complex which comprises at least six sibling phylogenetic species: *S. pallida, S. brasiliensis, S. globosa, S. luriei, S. mexicana,* and *S. schenckii* [1, 2]. Furthermore, *S. pallida,* *S. nivea*, and *S. albicans* were proposed to be synonyms since all the three species showed a considerably high genetic similarity [3]. Among the species of *Sporothrix* complex, there are differences in the geographical distributions. *S. brasiliensis* is restricted geographically to Brazil, and *S. mexicana* to Mexico [1]. Conversely, *S. globosa* is a widespread species found up to now in UK, Spain, Italy, China, Japan, USA, India, Mexico, Guatemala, Colombia, and more recently Brazil [1, 4, 5].

*Sporothrix globosa* species in China, which have been reported in the literature, are isolates from wheat, reed, and soil [6]. Which *Sporothrix* species (of clinical origin) being prevalent in China is still unknown to us. Northeast China including Heilongjiang, Jilin, and Liaoning provinces is the most endemic region [7–9] where sporadic cases have occurred for many decades, particularly in rural areas, with a high incidence in autumn and winter when the chances of contact with cornstalks increase. In recent years, small outbreaks have occurred in Jilin province with a significant increase in sporotrichosis.

The aim of this study was to investigate phylogenetic species of the *Sporothrix* complex in these areas and their morphological and physiological features, and to determine whether different clinical forms are associated with different species and whether outbreaks in Jilin province are caused by more virulent species than sporadic cases in Heilongjiang and Liaoning provinces.

## Materials and methods

### Fungal isolates

Seventy-four clinical isolates from human sporotrichosis patients were included in this study. All isolates were identified as *S. schenckii* by traditional morphological identification methods [3]. Forty-two of the isolates (SHJU_1–40_/FHJU_1_/FHJU_2_) were from Jilin province, ten of the isolates (HMU_1–10_) were from Heilongjiang province, and the remainder (DMU_1–22_) were from Liaoning province. The cases from Heilongjiang and Liaoning provinces were sporadic between 1998 and 2012, and the cases from Jilin province were outbreaks during 2010 and 2011. Of all isolates, three isolates (DMU1/FHJU1/FHJU2) were disseminated clinical form, 39 isolates (DMU_2–12_/HMU_1–5_/SHJU_18–40_) were lymphocutaneous clinical form, and 32 isolates (DMU_13–22_/HMU_6–10_/SHJU_1–17_) were fixed cutaneous clinical form. AM398393/AM398392/AM398396/AM747302/AM116899/AM117437/AM399018/AM490354/AM399004/AM399002/GU456632/AM116908/AM398994/AM490358/AM399005/AM399015 were also included in phylogenesis analysis (Table 1).

**Table 1:** Isolate, origin, clinical type, species, and GenBank accession numbers of *Sporothrix* isolates used in this study

Isolates^a^	Origin	Clinical type	Species^b^	GenBank
DMU1	Clinical, Liaoning, China	Disseminated	*S. globosa*	KC121564^c^
DMU2	Clinical, Liaoning, China	Lymphocutaneous	*S. globosa*	KC190222^c^
DMU3–DMU12	Clinical, Liaoning, China	Lymphocutaneous	*S. globosa*	This study
DMU13–DMU21	Clinical, Liaoning, China	Fixed	*S. globosa*	This study
DMU22	Clinical, Liaoning, China	Fixed	*S. globosa*	KC190217^c^
HMU1	Clinical, Heilongjiang, China	Lymphocutaneous	*S. globosa*	KC121565^c^
HMU2–HMU5	Clinical, Heilongjiang, China	Lymphocutaneous	*S. globosa*	This study
HMU6–HMU7	Clinical, Heilongjiang, China	Fixed	*S. globosa*	This study
HMU8	Clinical, Heilongjiang, China	Fixed	*S. globosa*	KC190221^c^
HMU9–HMU10	Clinical, Heilongjiang, China	Fixed	*S. globosa*	This study
FHJU1	Clinical, Jilin, China	Disseminated	*S. globosa*	KC121566^c^
FHJU2	Clinical, Jilin, China	Disseminated	*S. globosa*	KC190220^c^
SHJU1	Clinical, Jilin, China	Fixed	*S. globosa*	KC121567^c^
SHJU2	Clinical, Jilin, China	Fixed	*S. globosa*	KC190218^c^
SHJU3–SHJU17	Clinical, Jilin, China	Fixed	*S. globosa*	This study
SHJU18–SHJU39	Clinical, Jilin, China	Lymphocutaneous	*S.globosa*	This study
SHJU40	Clinical, Jilin, China	Lymphocutaneous	*S. globosa*	KC190219^c^
CBS120342	Environmental, Mexico		*S. mexicana*	AM398392
CBS120341^T^	Environmental, Mexico		*S. mexicana*	AM398393
CBS302.73^T^	Environmental, UK		*S. pallida*	AM398396
ATCC18616	Clinical, South Africa	NK	*S. luriei*	AM747302
CBS120339^T^	Clinical, Brazil	NK	*S. brasiliensis*	AM116899
CBS359.36^T^	Clinical, USA	NK	*S. schenckii*	AM117437
CBS120340^T^	Clinical, Spain	NK	*S. globosa*	AM116908
KMU4200	Reed leaves, China		*S. globosa*	AM399004
KMU4208	Cornstalks, China		*S. globosa*	AM399002
KMU4210	Soil, China		*S. globosa*	AM399005
CBS292.55^T^	Clinical, UK	NK	*S. globosa*	AM490354
IHEM4178	Clinical, Italy	NK	*S. globosa*	AM399018
IPEC27135	Clinical, Brazil	Lymphocutaneous	*S. globosa*	GU456632
FMR9020	Clinical, Japan	NK	*S. globosa*	AM398994
MCCL220029	Clinical, India	NK	*S. globosa*	AM490358
UTHSC04-1485	Clinical, USA	NK	*S. globosa*	AM399015

^a^Abbreviations: *DMU* The 1st Affiliated Hospital of Dalian Medical University; *HMU* The Second Hospital of Harbin Medical University; *FHJU* The First Hospital of Jilin University; *SHJU* The Second Hospital of Jilin University; *CBS* Centraalbureau voor Schimmelcultures, Utrecht, the Netherlands; *ATCC* American Type Culture Collection; *KMU* Kanazawa Medical University, Ishikawa, Japan; *IHEM, BCCM/IHEM* Biomedical Fungi and Yeasts Collection, Belgium; *IPEC* Instituto de Pesquisa Clínica Evandro Chagas, Fiocruz, Brazil; *FMR* Facultat de Medicina i Ciències de la Salut, Reus, Spain; *MCCL* Mycology Culture Collection Laboratory, Postgraduate Institute of Medical Education and Research, Chandigarh, India; *UTHSC* Fungus Testing Laboratory, University of Texas Health Science Center; ^T^ type strain; *NK* not known

^b^Identification based on calmodulin gene analysis

^c^The sequence was obtained in this study

### Morphological studies

In order to study macroscopic features and thermotolerance (10), all isolates were subcultured on Potato Dextrose Agar (PDA-Difco™ Becton, Dickinson and Company/Sparks, MD21152 USA) plates and incubated at various temperatures (30, 35, 37 °C) in the dark for 3 weeks. The petri dishes were centrally inoculated with 10 µl of the conidial suspension which was adjusted to 2.0–2.2 McFarland unit for each isolate, dried fully, and then placed upside down. The colony diameters (in mm) were measured after 21 days of incubation. The microscopic features were determined primarily from slide cultures made on Corn Meal Agar (CMA-Oxoid Ltd, Basingstoke, Hampshire, England) after 10–12 days of incubation at 30 °C. Coverslips were mounted in lactic acid and examined under a light microscope (Leica DM5000, German). The widths and lengths of the conidia were measured for each isolate. Dimorphism was demonstrated by conversion to the yeast-like form on Brain Heart Infusion (BHI-Bacto™ Becton, Dickinson and Company/Sparks, MD21152 USA) agar medium for 7 days at 37 °C.

### Physiologic studies

Carbohydrate assimilation tests were performed using freshly prepared yeast nitrogen base (YNB) medium (Difco™ Becton, Dickinson and Company/Sparks, MD21152 USA) and tested for dextrose, sucrose, and raffinose according to methods described previously [1]. Cultures on YNB supplemented with dextrose were used as a positive control for growth, and YNB without carbohydrates was used as a negative control. Experiments were performed twice. The tests were performed in 96-well microplates. Microplates were read after 5 days of incubation at 30 °C.

### Molecular identification

Genomic DNA was extracted and purified from *Sporothrix* spp. mycelial phase by phenol/chloroform/isoamyl alcohol method with lysis step for 20 min with glass beads [11]. Amplification of the partial calmodulin-encoding (CAL) gene was performed [12] with degenerated primers CL1-GA(GA)T(AT)CAAGGAGGCCTTCTC and CL2A–TTTTTGCATCATGAGTTGGAC as described by O’Donnell et al. [13]. Automated sequencing was done at Sangon Biotech (Shanghai, China) with the same primers used for PCR.

The sequences of all our isolates were compared by BLAST (http://www.ncbi.nlm.nih.gov/blast) with several published *Sporothrix*-calmodulin-related sequences available from NCBI GenBank (AM398393/AM398392/AM398396/AM747302/AM116899/AM117437/AM399018/AM490354/AM399004/AM399002/GU456632/AM116908/AM398994/AM490358/AM399005/AM399015) (Table 1). Computer-assisted multiple sequence comparisons were made using ClustalW algorithm implemented in MEGA4 software [14]. The multiple nucleotide sequence alignment was inspected, visually adjusted, and subsequently was used for neighbor-joining analysis performed using MEGA4 software [14], and confidence was estimated using 1,000 rounds of bootstrapping.

Ten representative nucleotide sequences: KC121564 (Isolate DMU1), KC121565 (Isolate HMU1), KC121566 (Isolate FHJU1), KC121567 (Isolate SHJU1), KC190217 (Isolate DMU22), KC190218 (Isolate SHJU2), KC190219 (Isolate SHJU40), KC190220 (Isolate FHJU2), KC190221 (Isolate HMU8), and KC190222 (Isolate DMU2) have also been submitted to GenBank database.

## Results

### Morphological characterization

**Fig. 1:**
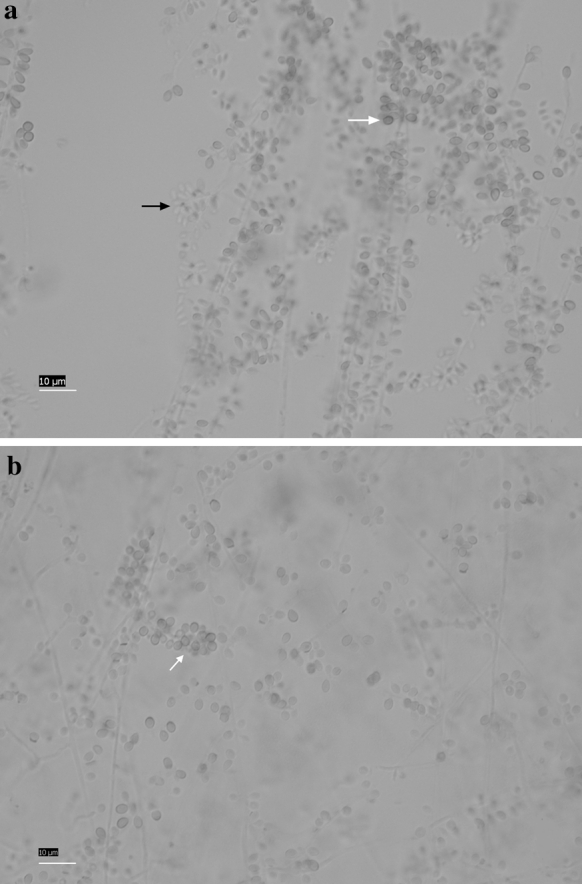
Morphology of conidia of the *S. globosa*. **a** *Black arrow* subhyaline, obovoidal conidia in sympodial conidiophores, *white arrow* dark, globose, and sessile conidia connected individually. **b** *White arrow* dark, globose, and sessile conidia connected individually. *Bars* 10 μm

**Fig. 2:**
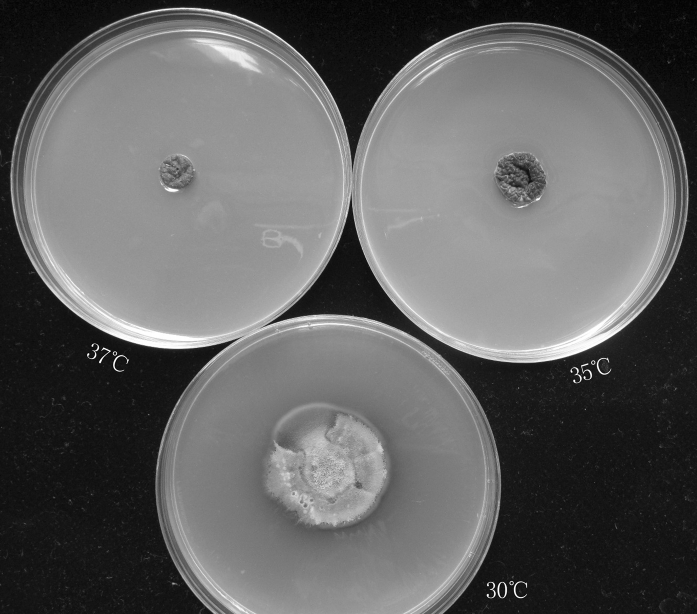
Colony of *S. globosa* developed on PDA at 30, 35 and 37 °C, respectively, in 21 days

The macroscopic morphologies of all isolates were similar. After 21 days of incubation, colonies were pale orange to gray-orange on PDA with many wrinkles. All isolates showed temperature dimorphism with the typical cigar-shaped form of the yeast-like cells on brain heart infusion (BHI) agar medium for 7 days at 37 °C. All isolates when cultured on corn meal agar for 12 days at 30 °C developed interim or terminal conidia in sympodial conidiophores along the hyphae. These conidia were hyaline to subhyaline, obovoidal or pear-shaped. In addition, sessile conidia were also observed, which were brown to dark brown, thick-walled, and connected individually throughout the hyphae, predominantly globose to subglobose, and 2.3–3.4 µm long by 1.9–2.8 µm wide (Fig. 1). After incubation, respectively, at 30, 35, and 37 °C in the dark for 3 weeks, the best fungal growth was observed at 30 °C, and the colonies attained a diameter of 25–45 mm with the mean colony diameter of 34 mm. Colony growth at 35 °C was lower than that observed at 30 °C, with 12–15 mm diameter and mean 13 mm diameter. At 37 °C, most isolates showed restricted growth (up to mean 9 mm in diameter) (Fig. 2), with the exception of three isolates (SHJU28, DMU3, DMU20) which were unable to grow at 37 °C.

### Physiologic characterization

Carbohydrate assimilation tests were run in double and presented the following results: all isolates assimilated dextrose and sucrose, and were unable to assimilate raffinose, and none was able to grow on controls without carbohydrates.

### Molecular analysis

**Fig. 3:**
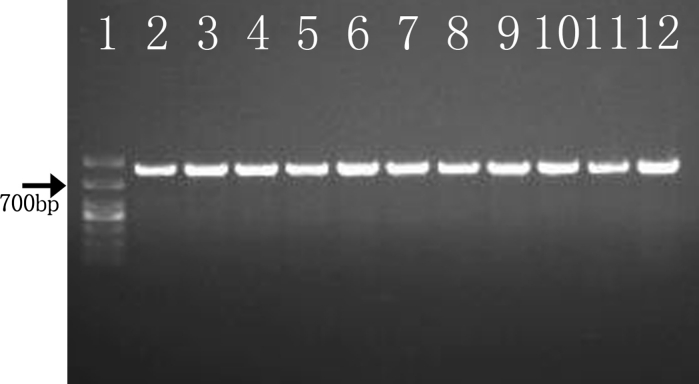
PCR product based on the partial calmodulin gene sequence with CL1-CL2A primers pair. *Lane 1* DL1000 marker-Takara; *Lanes 2–12* *Sporothrix * isolates: DMU1, DMU2, DMU22, HMU1, HMU2, HMU7, FHJU1, FHJU2, SHJU1, SHJU2, and SHJU40, respectively

**Fig. 4:**
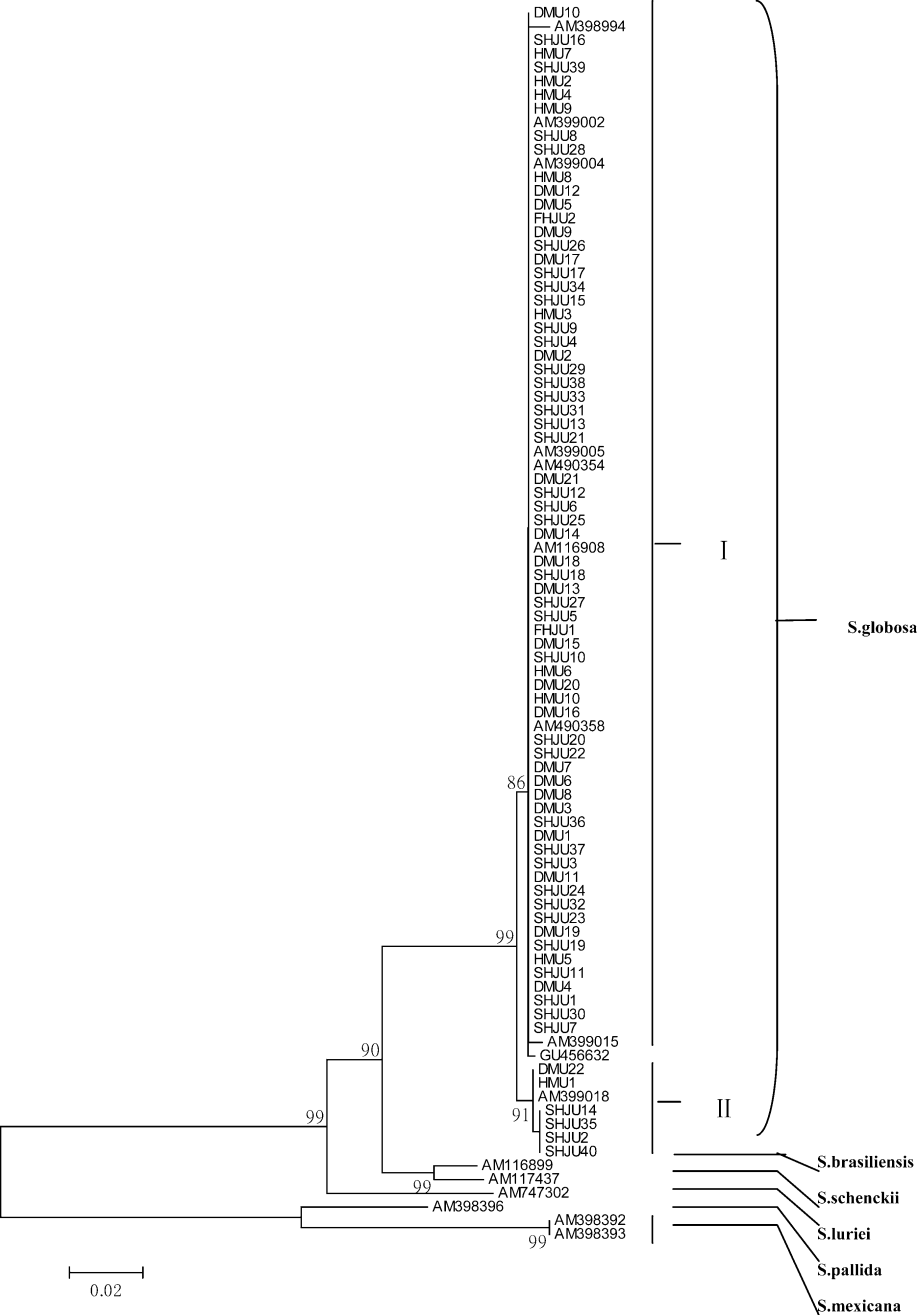
Phylogenetic tree generated by neighbor-joining analysis using partial nucleotide sequences of the calmodulin-encoding gene. Bootstrap support values above 85 % are indicated at the nodes

The primers CL1 and CL2A were used to amplify a fragment of approximately 770 base pairs (bp) of the CAL gene (Fig. 3). The sequences blast revealed all isolates had a high level of sequence similarity with previously published *S. globosa* strains listed in Table 1 (99–100 %). The phylogenetic tree of the CAL locus analyzed by neighbor joining—NJ method—revealed six well-defined and supported groups (Fig. 4) as previously described [6]. All clinical isolates originated in Northeast China in this study were clustered with group *S. globosa* (AM116908 and AM399018). Furthermore, our results showed that *S. globosa* could be further subdivided into two sub-clades: 68 of our isolates were clustered with AM116908, and the other six isolates were clustered with AM399018.

## Discussion

Our phylogenetic analysis showed that seventy-four clinical isolates originated in Northeast China were entirely clustered with *S. globosa*. Moreover, these isolates were divided into two highly supported sub-clades (*S. globosa* I and *S. globosa* II). *S. globosa* I grouped sixty-eight Chinese clinical isolates, three previously published Chinese environmental isolates (AM399002, AM399005, and AM399004), the type strain of *S. globosa* (AM116908 from Spain), and some isolates from the USA (AM399015), India (AM490358), Japan (AM398994), Brazil (GU456632), UK (AM490354), while *S. globosa* II included six Chinese clinical isolates and the isolate from Italy (AM399018). *S. globosa* I was significantly more frequent than *S. globosa* II. The division of *S. globosa* was not related to geography, since the main clade of our clinical isolates, *S. globosa* I was more closely related to isolates from USA, Japan, and Brazil than to the other Chinese clinical isolates of *S. globosa* II. Sixty-eight Chinese clinical isolates were closely related to AM399002, AM399004, and AM399005, which were isolated from environment of Northeast China by Ishizaki and Xuezhu Jin [15]. Among them, AM399002 was from cornstalks, while AM399004 was from reed leaves and AM399005 was from soil [15], which indicated that most clinical isolates in Northeast China originated from autochthonous environment. Only six isolates were related to AM399018, which was isolated from Italy by Viviani in 1986 [16]. This suggested that a small amount of isolates in Northeast China were allochthonous. There was no association between the clinical form of sporotrichosis and the division of *S*. *globosa*. The origins of *S. globosa* I isolates in this study were fixed cutaneous (*n* = 29), lymphocutaneous (*n* = 36), and disseminated cutaneous (*n* = 3). *S. globosa* II isolates in this study were from fixed cutaneous (*n* = 3) and lymphocutaneous (*n* = 3) cases.

Although these clinical isolates were divided into two groups, they showed the same morphological and physiological features. They produced not only obovoidal, hyaline, sympodial conidia but also globose to subglobose, pigmented, sessile conidia. They were able to assimilate sucrose and unable to assimilate raffinose. The colonies of these isolates when grown on PDA attained a diameter of 25–45 mm at 30 °C in 21 days (not exceeding 50 mm). According to the Marimon’s key phenotypic features for species differentiation [1], the aforementioned phenotypic aspects are characteristic of *S. globosa.* However, we also noticed an important discrepancy with the results reported by Marimon et al. [1] with regard to the ability of *S. globosa* isolates to grow at 37 °C. Marimon et al. proposed *S. globosa* did not present growth at 37 °C. By contrast, most of our *S. globosa* isolates presented growth at 37 °C, attaining 9 mm of colony diameter on PDA at 37 °C in 21 days. Nevertheless, Marimon et al. related four exceptions, which exhibited very restricted growth (up to 2 mm in diameter in 21 days). Moreover, the first *S. globosa* isolate in Brazil identified by Oliveira et al. [5] also presented growth at 37 °C, attaining 7 mm of colony diameter. Consequently, disagreement in thermotolerance suggested that there may be some variation within this species. It seemed that the capacity for growth at 37 °C was not helpful for species differentiation. The reason for the difference in diameter at 37 °C may be that we inoculated 10 μl of conidial suspension, while both Marimon and Oliveira inoculated pieces of the fungus that were approximately 1 mm in diameter. In addition, Kwon-Chung [17] discovered that strains causing fixed cutaneous sporotrichosis grow best at 35 °C, while those causing lymphocutaneous form grow at both 37 and 35 °C. However, in our study, the isolates which were thermotolerant at 37 °C could be obtained from fixed cutaneous, lymphocutaneous, or disseminated sporotrichosis. Our observations were similar to those of Mehta et al. [18] who observed that isolates from both fixed and lymphocutaneous types grew well at 37 °C. These phenomena further hinted that difference in thermotolerance may be related to individual variation.

Based on the phenotypic and genetic analysis, we found that all clinical isolates studied belonged to the same species, i.e., *S. globosa*, independent of geographical regions and clinical forms of sporotrichosis, which suggested it was not *Sporothrix* species that determined types of clinical presentation. Our previous studies [19] showed that the isolate (Sp98-12-1, i.e. DMU1) from disseminated sporotrichosis presented 10-bp deletion in the ribosomal nontranscribed spacer (NTS) region and higher virulence compared to the isolate (D1, i.e. DMU15) from fixed sporotrichosis, which further suggested from the gene level that there may be some variation within this species. It appears that the strain variation in genotypes and virulence as well as immune status of the host may contribute to disseminated type of sporotrichosis. We also drew a conclusion that outbreaks of sporotrichosis in Jilin province over the past 2 years were caused by the same species as the sporadic cases in Heilongjiang and Liaoning provinces, which could be explained by the fact that in Northeast China, wide contact with contaminated cornstalks led to high incidence. The people living in rural areas accounted for a considerable proportion of sporotrichosis patients, who were used to stacking cornstalks for use in cooking or heating. Isolation of *S*. *schenckii* from cornstalks of Northeast China has been reported [15], which proves to be *S*. *globosa* now [1]. Over time, fungal growth on this material increases as the cornstalks decay and become the source of contamination. Outbreaks might be associated with increased chances of contact in autumn and winter or increased *Sporothrix* quantity in the environment, which was also confirmed by our results that most clinical isolates were clustered with Chinese environmental isolates.
